# Diagnostic value of FIB-4 for liver fibrosis in patients with hepatitis B: a meta-analysis of diagnostic test

**DOI:** 10.18632/oncotarget.14430

**Published:** 2017-01-02

**Authors:** Zhi Yin, Jin Zou, Qiongxuan Li, Lizhang Chen

**Affiliations:** ^1^ Department of Epidemiology and Health Statistics, Xiangya School of Public Health, Central South University, Changsha, Hunan Province 410078, China; ^2^ Division of Recruitment and Employment, University of South China, Hengyang, Hunan Province 421001, China; ^3^ Department of Cardiology, The First Affiliated Hospital of University of South China, Hengyang, Hunan Province 421001, China

**Keywords:** liver fibrosis, hepatitis B, FIB-4, meta-analysis

## Abstract

This study is aimed at evaluating the diagnostic value of FIB-4 for liver fibrosis in patients with hepatitis B through a meta-analysis of diagnostic test. We conducted a comprehensive search in the Pubmed, Embase, Web of Science, and Chinese National Knowledge Infrastructure before October 31, 2016. Stata 14.0 software was used for calculation and statistical analyses. We used the sensitivity, specificity, positive and negative likelihood ratio (PLR, NLR), diagnostic odds ratio (DOR) and 95% confidence intervals (CIs) to evaluate the diagnostic value of FIB-4 for liver fibrosis in patients with hepatitis B. Twenty-six studies were included in the final analyses, with a total of 8274 individuals. The pooled parameters are calculated from all studies: sensitivity of 0.69 (95%CI:0.63-0.75), specificity of 0.81 (95%CI: 0.73-0.87), PLR of 3.63 (95%CI:2.66-4.94), NLR of 0.38 (95%CI:0.32-0.44), DOR of 9.57 (95%CI: 6.67-13.74), and area under the curve (AUC) of 0.80 (95%CI: 0.76-0.83). We also conducted subgroup based on the range of cut-off values. Results from subgroup analysis showed that cut-off was the source of heterogeneity in the present study. The sensitivity and specificity of cut-off>2 were 0.69 and 0.95 with the AUC of 0.90 (95%CI: 0.87-0.92). The overall diagnostic value of FIB-4 is not very high for liver fibrosis in patients with hepatitis B. However, the diagnostic value is affected by the cut-off value. FIB-4 has relatively high diagnostic value for detecting liver fibrosis in patients with hepatitis B when the diagnostic threshold value is more than 2.0.

## INTRODUCTION

It is reported that 24 billion populations have been exposed to hepatitis B virus (HBV). Three point five people are patients with chronic hepatitis B virus, and 75 million population died of hepatic failure, liver cirrhosis, liver cancer caused by HBV infection. Hepatitis B has become one of important public health issues [1]. Early detection and identification of liver fibrosis to prevent progression to cirrhosis is the goal of treatment in in patients with hepatitis B. In the past decades, liver biopsy has been considered as the gold standard for determining liver fibrosis. However, this invasive operation is related to several disadvantages including sampling error and some variations [2]. These application limitations result in developing dependable, non-invasive methods to detect the stage of fibrosis in patients with hepatitis B. Recently, research has focused on the development of noninvasive tests for the evaluation of liver fibrosis; serum-based tests have attracted the maximum attention One of the previous methods is Fibro test which was conducted in patients with hepatitis C. Subsequent models: aspartate aminotransferase to platelet ratio index (APRI), Forn's score, ELF-score, Hepascore Fibrometer have been studied worldwide [3]. The use of noninvasive indices such as the aspartate aminotransferase to platelet ratio index (APRI), the fibrosis index based on the four factors (FIB-4: the FIB-4—a test derived from the Apricot database, which produces interesting results using the following formula: age (years) × AST [U/L]/(platelets [10^9^/L] × (ALT [U/L])^1/2^) score [4], and the Forn's index has been suggested as a method to assess liver fibrosis in patients with chronic liver diseases [5].

The non-invasive diagnostic methods have become a new field. There are studies reported that the diagnostic accuracy of FIB-4 for liver fibrosis in patients with hepatitis B. However, the diagnostic ability from different study is obviously different, which may be affected by some limitations such as sampling error, inter-and intra-observer variations. Considering the limitation of single study, we try to conduct this meta-analysis based on more study samples and statistics, aiming to acquire the diagnostic efficiency of FIB-4 for liver fibrosis in patients with hepatitis B more accurately.

## RESULTS

### Study selection and study characteristics

**Figure 1:**
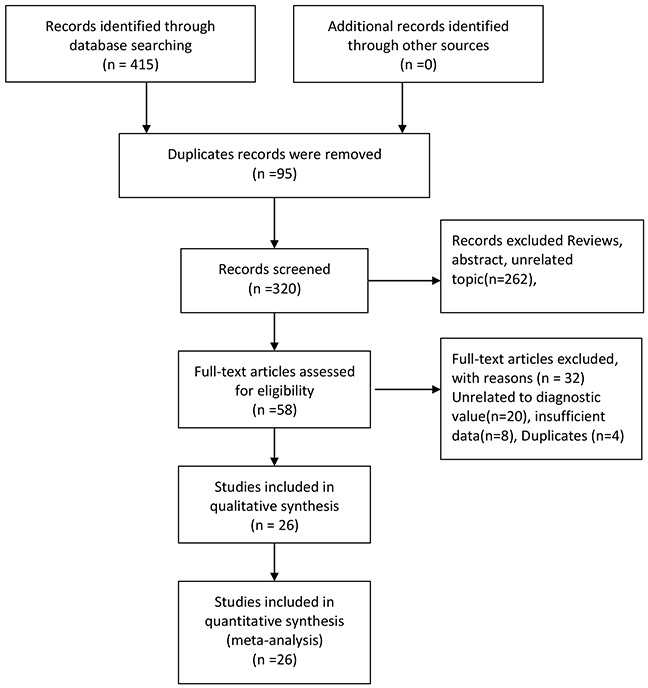
Flow diagram of studies selection process

The selection flow of literature search was presented in Figure 1. Our initial search obtained 15 records. 95 duplicates records were removed, and 262 records were excluded after reviewing titles, abstract and topic. 58 full-text articles were assessed for eligibility. With a further work on reading full texts of 58 articles, we removed 20 records unrelated to diagnostic value, and 4 duplicates. Similarly, 8 articles were excluded for unable to provided sufficient data. Finally, 26 articles were entered into the final qualitative and quantitative analyses [3, 5–29].

In Table 1, we summarized the characteristics of the 26 included articles in the meta-analysis of FIB-4 for liver fibrosis. The publication year of 26 studies ranged from 2009 to 2015. The studies were from different countries all over the world, and included 8274 individuals. Of the 26 studies, 3 studies are prospective design and 23 are retrospective. Most of them are multicenter studies. The sample size varied from 52 to 2411. Among the 26 studies, 18 studies were conducted in Asian (China, India, Korea), 4 in Caucasian population, 3 in Central East, and 1 in African population. The four-fold table data was presented in Table 1.

**Table 1:** Characteristics of the included studies in the meta-analysis

No.	Author	Year	Region	Mean age(y)	Sample size	Study design	Study population	Length of tissue	Cut off	TP	FP	FN	TN	Score of Quality
1	Li	2013	USA	45	284	Retrospective	Multicenter	-	5.17	19	1	32	232	13
2	Koksal	2015	USA	43	229	Retrospective	Multicenter	-	1.45	98	5	94	32	12
3	Erdogan	2013	Turkey	41	221	Retrospective	Single center	-	1.02	53	63	15	90	12
4	Ucar	2013	Turkey	45	73	Retrospective	Single center	-	1.09	29	12	12	20	13
5	Shrivastava	2013	India	30	52	Retrospective	Single center	15-20	2.50	2	2	4	44	14
6	Basar	2013	Turkey	45	76	Prospective	Single center	>10	1.09	37	6	14	19	14
7	Seto	2011	H.K.	38	237	Retrospective	Single center	>15	1.45	40	41	37	119	14
8	Sebastiani	2011	French	47	2411	Retrospective	Multicenter	-	1.45	784	455	328	844	14
9	Kim	2010	Korea	39	668	Retrospective	Single center	>15	1.00	301	92	29	246	14
10	Bonnard	2010	Africa	35	59	Prospective	Single center	-	0.80	30	7	11	11	12
11	Mallet	2009	French	42	138	Retrospective	Multicenter	17.6	1.45	29	26	12	71	13
12	Wu	2010	China	33	78	Retrospective	Multicenter	>15	1.45	20	7	12	39	14
13	Liu	2011	China	32	623	Retrospective	Multicenter	>15	1.10	158	130	57	278	13
14	Zhu	2011	China	37	175	Retrospective	Multicenter	>15	1.70	57	15	22	81	12
15	Wu	2012	China	33	482	Retrospective	Multicenter	>15	1.57	189	66	81	146	13
16	Zhu	2012	China	42	159	Prospective	Single center	>15	4.90	91	10	13	45	12
17	Chen	2013	China	40	148	Retrospective	Single center	>15	1.45	27	29	13	79	13
18	Wang	2013	China	34	231	Retrospective	Single center	>15	1.45	37	24	31	139	11
19	Wang	2013	China	37	149	Retrospective	Multicenter	>10	1.45	60	21	29	39	12
20	Xun	2013	China	31	197	Prospective	Single center	>15	1.00	80	26	32	59	13
21	Zeng	2013	China	36	198	Retrospective	Single center	15-20	31.61	25	35	13	125	12
22	Liu	2014	China	38	111	Retrospective	Single center	16.67	2.29	11	28	1	74	12
23	Xu	2015	China	36	446	Retrospective	Single center	>16	1.07	160	84	59	143	11
24	Li	2013	China	45	284	Retrospective	Multicenter	-	5.17	19	1	32	232	11
25	Ji	2011	China	36	313	Retrospective	Single center	>20	2.96	44	19	6	244	11
26	Li	2015	China	38	232	Retrospective	Single center	-	1.58	66	46	20	152	11

### Quality assessment

The quality score of each study was presented in Table 1. The score of each study was more than 11 points. All the included studies received moderately high scores from the QUADAS-2 quality assessments.

### Pooled diagnostic values

**Figure 2:**
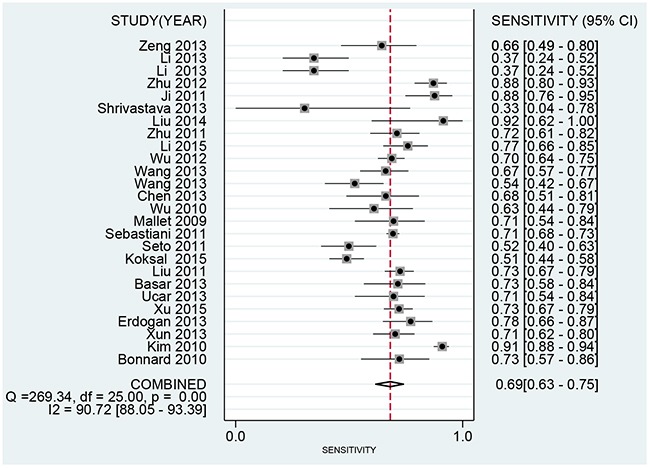
Forest plot of pooled sensitivity of FIB-4 for liver fibrosis in patients with hepatitis B

**Figure 3:**
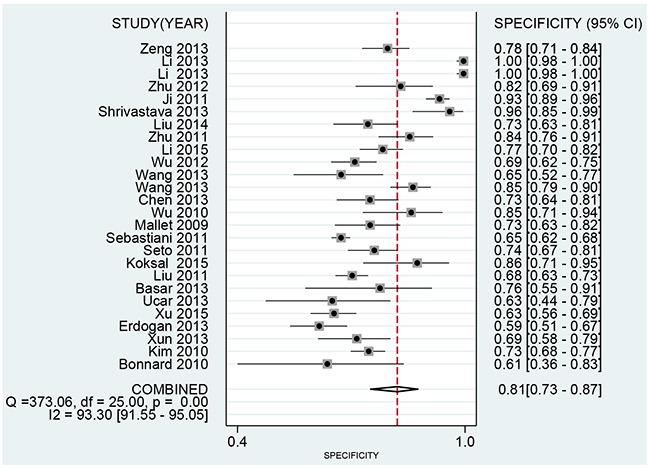
Forest plot of pooled specificity of FIB-4 for liver fibrosis in patients with hepatitis B

**Figure 4:**
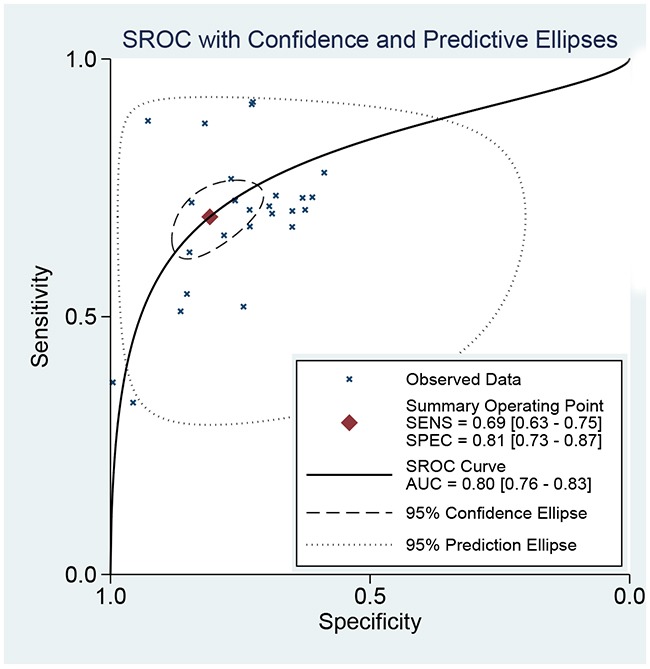
The SROC curve of FIB-4 for liver fibrosis in patients with hepatitis B

**Figure 5:**
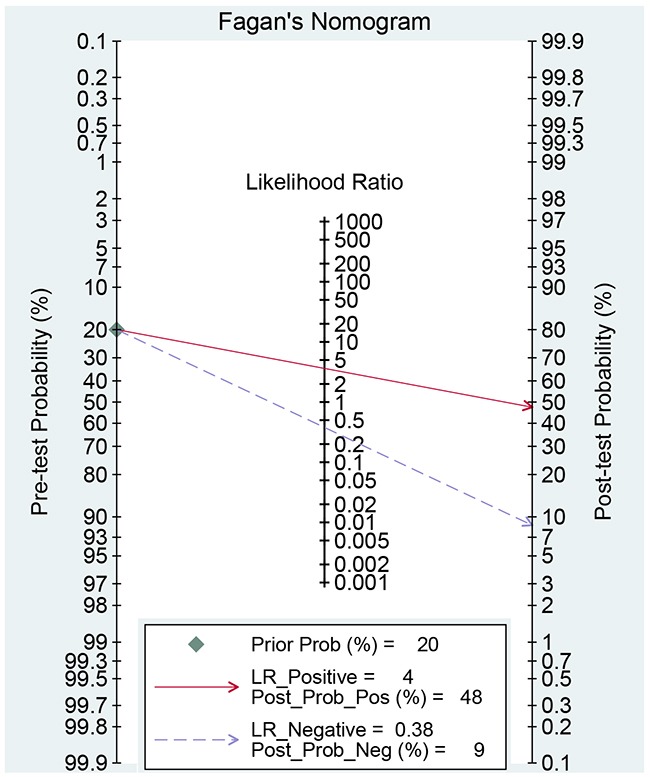
Fagan diagram evaluating the overall diagnostic value of FIB-4 for liver fibrosis in patients with hepatitis B

We use the random effect model to pool the sensitivity and specificity because the *I^2^* values were more than 50%. The pooled diagnostic values of FIB-4 for detecting liver fibrosis in patients with hepatitis B were presented in Table 2. The overall pooled sensitivity and specificity were 0.69 (95%CI:0.63-0.75, Figure 2) and 0.81 (95%CI: 0.73-0.87, Figure 3). The pooled PLR was 3.63 (95%CI:2.66-4.94), NLR was 0.38 (95%CI:0.32-0.44), and DOR was 9.57 (95%CI: 6.67-13.74). The overall SROC curve was shown in Figure 4, and AUC was 0.81 (95%CI: 0.77-0.84). Fagan plot was shown in Figure 5. The prior probability was 20%, and the post-test probability was 48% of LR-positive, and 9% of LR-negative. The diagnostic accuracy of FIB-4 for detecting liver fibrosis in patients with hepatitis B was not very high.

**Table 2:** Summary estimated of diagnostic performance of FIB-4 for liver fibrosis

Category	SEN (95%CI)	SPE (95%CI)	PLR (95%CI)	NLR (95%CI)	DOR (95%CI)	AUC (95%CI)
Overall	0.69[0.63-0.75]	0.81[0.73-0.87]	3.63[2.66-4.94]	0.38[0.32-0.44]	9.57[6.67-13.74]	0.80[0.76-0.83]
0.8-1.1	0.77[0.70-0.82]	0.66[0.63-0.70]	2.29[1.95-2.68]	0.35[0.26-0.47]	6.52[4.18-10.19]	0.72[0.68-0.76]
1.2-2.0	0.65[0.60-0.70]	0.76[0.711-0.81]	2.72[2.28-3.24]	0.46[0.41-0.52]	5.88[4.59-7.55]	0.76[0.72-0.79]
>2	0.69[0.60-0.84]	0.95[0.83-0.99]	12.89[4.47-37.18]	0.33[0.19-0.57]	39.17[16.13–95.13]	0.90[0.87-0.92]

We subgroup analyses based on the range of cut-off values. Results from subgroup analysis showed that cut-off was the source of heterogeneity in the present study. The sensitivity and specificity of cut-off>2 were 0.69 (95%CI: 0.60-0.84) and 0.95 (95%CI: 0.83-0.99) with the AUC of 0.90 (95%CI: 0.87-0.92). The pooled diagnostic values other two cut-off range was presented in Table 2.

### Sensitivity analysis and publication bias

**Figure 6:**
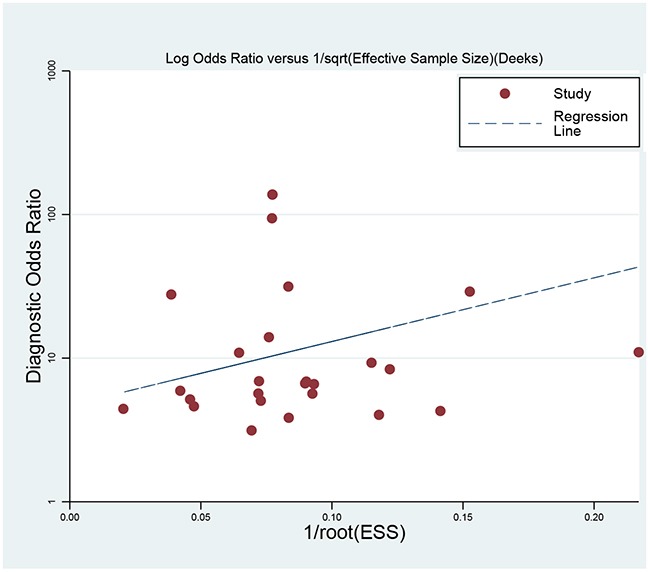
Deek's funnel plot to evaluate the publication bias

Sensitivity analysis was conducted by sequentially excluding some special studies (with small sample size, bigger cut-off value) [20, 27]. The pooled results did not alter, indicating the results were stable. The Deek's plot shows there was no publication bias (t=1.670, *P*=0.107, Figure 6).

## DISCUSSION

With the increasing popularity of hepatitis B over the world, it is needed for effective and convenient diagnostic methods. In the past decades, the arise of non-invasive and new methods in detecting liver fibrosis had developed rapidly. Among all kinds of examination methods, more and more researchers paid attention on combined diagnostic index. FIB-4, one of the novel methods in detecting liver fibrosis, attracted more attention and was widely explored for its role in patients with hepatitis B [5].

In this meta-analysis, we found FIB-4 achieved the overall pooled sensitivity of 0.69 (95%CI:0.63-0.75) and specificity 0.81 (95%CI: 0.73-0.87), and AUC was 0.81 (95%CI: 0.77-0.84). The results showed that the diagnostic accuracy may not be high enough as expected. However, FIB-4 still achieves almost same level diagnostic value with some invasive methods [21]. The DOR represents the value that combines sensitivity and specificity that ranges from 0 to infinity, with higher value meaning better diagnostic ability. Our results show the DOR was 9.57 (6.67-13.74), suggesting the overall pooled accuracy was not high. The pooled PLR of 3.63 means that patients with liver fibrosis have approximately a 3.63-fold higher chance of being FIB-4 positive compared with individuals without liver fibrosis. The pooled NLR was 0.38, indicating that the probability of patients having liver fibrosis is 38% if the FIB-4 was negative. According to the criteria, the accuracy is considered to be high when PLR>10 and NLR<0.1. The present result did arrive the standard, suggesting that FIB-4 had sub-optimal accuracy for clinical purpose [30]. However, FIB-4 has relatively high diagnostic value for detecting liver fibrosis in patients with hepatitis B when the diagnostic threshold value was more than 2.0. This result indicates that FIB had higher accuracy for developed liver fibrosis in patients with hepatitis B. Considered the diagnostic ability of other non-invasive methods, the advantage of FIB-4 is not outstanding in severe liver fibrosis stage. Previous studies reported that the aspartate transaminase/blood platelet index (APRI) and Fibro were also potential non-invasive methods. Previous meta-analysis suggested that AUCs of APRI and Fibro for detecting liver fibrosis were 0.75 and 0.87 [31, 32]. The diagnostic ability of FIB-4 is prior to APRI but less than Fibro. The FIB-4 was firstly applied in the diagnostic of hepatitis C. It is reported that the AUC of FIB-4 was 0.74 in patients with hepatitis C. The FIB-4 in hepatitis B was obviously higher than in the hepatitis C [33].

Our results found that the diagnostic of FIB-4 for liver fibrosis had many different kinds of cut-off values. It is pretty difficult for clinical practice. Therefore, this studies conducted subgroup analyses based the range of cut-off values. The diagnostic threshold value of FIB-4 achieves the highest AUC when the cut-off was more than 2. The corresponding AUC was 0.90 (95%CI: 0.87-0.92) with the sensitivity of 0.69 and the specificity of 0.95, indicating the diagnostic ability within this interval is higher than other range. However, there are still some studies with so high cut-off values that they can't be pooled in the meta-analysis. Xu et al used the 223.7 as the cut-off value of liver fibrosis in the patients with hepatitis B, which can't be included in the final analyses. More study with strict design, larger sample size, and multicenter are required to test the accuracy of FIB-4 and identify the proper cut-off value range.

We strictly follow the PRISM guidelines to conduct the meta-analysis [34]. But, there are still some limitations in the present meta-analysis. First, although we have tried our best to search relevant studies, we may neglect some studies non-published online. Second, there could exist some selection bias of population in the meta-analysis because the present studies only one African population, and most of studies are from Asian population. Third, the condition of study population included in the meta-analysis may have other diseases, which could overestimate or underestimate the diagnostic ability of FIB-4 for liver fibrosis. Four, the overall heterogeneity was high (*I^2^*: 88.0%-95%.0), we did not found the source of heterogeneity by subgroup. Finally, we did not consider the relation between sample quality and different cut-off values. Although our results presented that FIB-4 has relatively high diagnostic value for detecting liver fibrosis in patients with hepatitis B when the diagnostic threshold value was more than 2.0, the diagnostic ability of FIB-4 would be different in different progression of liver fibrosis such as liver cirrhosis. For different stage of liver fibrosis, it requires more studies and analyses to confirm the diagnostic value of FIB-4 in specific stage fibrosis.

## CONCLUSIONS

In conclusions, our analysis showed that the overall diagnostic value of FIB-4 may not be very high and have sub-optimal accuracy for liver fibrosis in patients with hepatitis B. However, the diagnostic value is affected by the cut-off value. FIB-4 has relatively high diagnostic value for detecting liver fibrosis in patients with hepatitis B when the diagnostic threshold value was more than 2.0. We expect further studies to confirm our analysis.

## MATERIALS AND METHODS

### Literature search

A systematic search was conducted for relevant articles published in the PubMed, Embase, Web of Science, and Chinese National Knowledge Infrastructure from inception to October 31, 2016. The following keywords are used: FIB-4, aspartate aminotransferase, AST, alanine aminotransferase, ALT, platelet, PLT, hepatitis B, liver fibrosis, and cirrhosis. The language was restricted in Chinese and English. We also retrieve the reference lists of relevant reviews to identify to additional studies.

### Selection criteria

The included studies must meet the following criteria: (1) all the patients with liver fibrosis must be diagnosed through the gold standard (liver biopsy). (2) studies provided diagnostic value of FIB-4 for liver fibrosis. (3) study must present sufficient data to allow calculation of the diagnostic value: True positive (TP), false positive (FP), false negative (FN), and true negative (TN). Duplicate publications, studies without qualified data, focused on other diseases, and letters, reviews, case reports and editorials were excluded.

### Data extraction

For each study included in the meta-analysis, the following information was extracted: the first author, publication year, region, mean age, sample size, study design, study population, length of tissue, cut-off value of diagnostic, four data (TP, FP, FN, TN). Two authors (ZJ and LQX) independently extract this information by using a standard excel sheet, and cross check the data. Any disputes were solved by the third investigator (CLZ).

### Quality evaluation

We used the Quality Assessment of Diagnostic Accuracy Studies 2 (QUADAS-2) to assess the quality of included studies. We used a quantitative method to assess the studies. The QUADAS-2 included 14 items [35]. Each key domain includes two sections: risk of bias and applicability. If answers to all signaling questions for a domain are ‘yes’, then we could judge the risk of bias is low. If any question is answered ‘no’, potential bias exists. Concerns about applicability are judged as ‘low’, ‘high’, or ‘unclear’. We defined ‘Yes’ as one scores.

### Statistical analysis

We used the Stata 14 software (StataCorp LP, College Station, TX, USA) to perform all statistical analyses. The heterogeneity within studies was evaluated by Q Test and *I^2^* test, and *I^2^*>50% presented the existence of heterogeneity [36]. The bivariate regression model was used to calculate the polled sensitivity, specificity, positive and negative likelihood ratios (PLRs and NLRs), diagnostic odds ratio (DOR) and their 95% confidence intervals (CIs) [37]. We also calculated the area under the receiver operator characteristic curve (SROC, AUC). The AUC ranged from 0 to 1, and an AUC of 1 represents the perfect discrimination ability, while an AUC<0.5 shows a poor diagnostic ability [38]. We also conducted a subgroup based on cut-off value. We used the Deek's funnel plot to assess the publication bias, and Fagan plots shows the relationship between the prior probability, the likelihood ration, and posterior test probability [39]. *P*<0.05 was considered to be significant.

## Abbreviations

APRI: Aspartate aminotransferase to platelet ratio index TP
FIB-4: Fibrosis index based on the 4 factor
AST: aspartate aminotransferase
ALT: alanine aminotransferase
PLT: platelet
QUADAS-2: Quality Assessment of Diagnostic Accuracy Studies 2
TP: true positives
FP: false positives
FN: false negatives
TN: true negatives
ROC: Receiver Operating Characteristic
SROC: summary receiver operator characteristic PLRs, positive likelihood ratios
NLRs: negative likelihood ratios
DORs: diagnostic odds ratios
CI: confidence intervals
